# The Treatment of Raynaud's Disease

**Published:** 1901-09-21

**Authors:** 


### THE TREATMENT OF RAYNAUD'S DISEASE.

In lecturing a little time ago at the London Hospital on the subject of that group of phenomena which is commonly spoken of as Raynaud's disease, Mr. Jonathan Hutchinson first pointed out the many causes by which interference with the circulation of the extremities might be so interfered with as to produce conditions which might be described, according to their variations, as local syncope, local asphyxia, or symmetrical gangrene of the extremeties. But he insisted upon the fact that, in order to constitute a typical resemblance to Raynaud's phenomena, the patient ought to be affected in a paroxysmal manner, that is to say that the symptoms ought to come and go. In regard to treatment, he said that if it seemed that the nervous system was very susceptible in a reflex manner to the influence of temperature, the chief method of treatment would be to protect the individual from cold, and in young children the main measure was to insist upon warm clothing, and if possible to take care that they lived in a warm climate. In his private practice, Mr. Hutchinson said, he always tried to send a patient who was liable to Raynaud's phenomena to winter in Egypt—not to a tropical but to a warm climate —and he could speak in clear terms as to the benefits derived by such patients from habitually spending the cold part of the year out of England. If that could not be done, there should be every precaution in the shape of warm clothing, to whatever extent might be necessary, to protect them from the injurious influence of external temperature. Then, if the case were one of scleriasis or morphœa, and the patient were an adult who could not go away from England during cold parts of the year—and this remark applied to all forms of gangrene of the extremities—the very best remedy for preventing undue susceptibility in regard to reflex influences and for maintaining the circulation in the extremities was opium. If purgatives be given in small quantities, such as from three to five drops of laudanum three or four times a day, with tonics, and careful attention to the liver, and these agree, other remedies may be useful, such as the moderate use of stimulants or the employment of galvanism; but, says Mr. Hutchinson, "my faith is in the use of tonics, the maintenance of the external temperature, and the exhibition of small doses of opium for a long time; it may be even extending to years."

